# Endometriosis and irritable bowel syndrome: A systematic review and meta-analyses

**DOI:** 10.3389/fmed.2022.914356

**Published:** 2022-07-25

**Authors:** Michelle Y. Nabi, Samal Nauhria, Morgan Reel, Simon Londono, Anisha Vasireddi, Mina Elmiry, Prakash V. A. K. Ramdass

**Affiliations:** ^1^Department of Public Health and Preventive Medicine, School of Medicine, St. George’s University, St. George’s, Grenada; ^2^Department of Pathology, School of Medicine, St. Matthew’s University, George Town, Cayman Islands

**Keywords:** irritable bowel syndrome, endometriosis, systematic review, meta-analyses, functional gastrointestinal disorders

## Abstract

**Objective:**

To estimate the pooled odds ratio of endometriosis and irritable bowel syndrome, and to estimate the pooled prevalence of irritable bowel syndrome in patients with endometriosis.

**Data sources:**

Using Cochrane Library, MEDLINE, Science Direct, ClinicalTrials.gov, Web of Science, and CINAHL, we conducted a systematic literature search through October 2021, using the key terms “endometriosis” and “irritable bowel syndrome.” Articles had to be published in English or Spanish. No restriction on geographical location was applied.

**Methods of study selection:**

The following eligibility criteria were applied: full-text original articles; human studies; studies that investigated the association between endometriosis and irritable bowel syndrome. Two investigators screened and reviewed the studies. A total of 1,776 studies were identified in 6 separate databases. After screening and applying the eligibility criteria, a total of 17 studies were included for analyses. The meta-analysis of association between endometriosis and irritable bowel syndrome included 11 studies, and the meta-analysis on the prevalence of irritable bowel syndrome in endometriosis included 6 studies.

**Tabulation, integration, and results:**

Overall 96,119 subjects were included in the main meta-analysis (11 studies) for endometriosis and irritable bowel syndrome, with 18,887 endometriosis patients and 77,171 controls. The odds of irritable bowel syndrome were approximately 3 times higher among patients with endometriosis compared with healthy controls (odds ratio 2.97; 95% confidence interval, 2.17 – 4.06). Similar results were obtained after subgroup analyses by endometriosis diagnosis, irritable bowel syndrome diagnostic criteria, and Newcastle-Ottawa Scale scores. Six studies reported prevalence rates of irritable bowel syndrome in women with endometriosis, ranging from 10.6 to 52%. The pooled prevalence of irritable bowel syndrome in women with endometriosis was 23.4% (95% confidence interval, 9.7 – 37.2).

**Conclusion:**

Patients with endometriosis have an approximately threefold increased risk of developing irritable bowel syndrome. Development and recent update of Rome criteria has evolved the diagnosis of IBS, potential bias should still be considered as there are no specific tests available for diagnosis.

**Systematic Review Registration:**

[https://www.crd.york.ac.uk/prospero/displa y_record.php?ID=CRD42018080611], identifier [CRD42018080611].

## Introduction

Endometriosis and irritable bowel syndrome (IBS) are two common medical conditions that markedly affect a substantial proportion of women and teenage girls, and even some menopausal women (1, 2). Even though they are two distinct conditions with different etiologies, a significant percentage of women experience both concurrently (3). Endometriosis, with an estimated worldwide prevalence ranging from 0.7 to 8.6%, (4) is characterized by the existence of endometrial-like tissue that has been disseminated beyond the uterine cavity. Patients with endometriosis commonly experience menstrual disturbance, infertility, abdominal and pelvic pain, and irregularities with bowel movements (5, 6).

Irritable bowel syndrome, which shares many clinical features with endometriosis, is a gastrointestinal disorder that primarily affects the large intestine, and is characterized by an array of symptoms such as alteration in bowel movements, abdominal discomfort, pain, and cramping (7). The prevalence of irritable bowel syndrome ranges from 0.4 (in India and Ghana) to 20.9% (in Singapore), with a pooled global prevalence of 5.9% (8). Moreover, approximately 61% of women and teenage girls are affected by irritable bowel syndrome (9).

Irritable bowel syndrome and endometriosis have a significant overlap in symptom presentation due to chronic inflammation thus leading to chronic pelvic pain (10). Endometriosis may even masquerade as irritable bowel syndrome in some patients (11). However, despite these similarities in clinical presentation, a recent nationwide study in the U.S. has shown that endometriosis increases the risk of irritable bowel syndrome approximately threefold (3). Possible explanations for this increased risk include chronic low grade inflammation resulting from mast cell activation, neuronal inflammation, leaky gut, and dysbiosis (12).

It is unclear if endometriosis is an independent risk factor for irritable bowel syndrome. The main purpose of this study was to quantify the association between endometriosis and irritable bowel syndrome, and to estimate the prevalence of irritable bowel syndrome in patients with endometriosis through pooled analysis.

## Materials and methods

### Sources

This systematic review and meta-analysis was conducted in accordance with the Preferred Reporting Items for Systematic Reviews and Meta-Analyses (PRISMA) guidelines and protocols (PRISMA-P) statement (13). The study protocol was registered in the PROSPERO database (University of York, United Kingdom).^1^ A systematic search of the following electronic databases was conducted to identify peer-reviewed literature from inception until October 2021: MEDLINE, Science Direct, ClinicalTrials.gov, Central Register of Controlled Trials, Cochrane Database of Systematic Reviews, CINAHL, and Web of Science. Key words or MeSH terms used were “irritable bowel syndrome” AND “endometriosis.”

### Study selection

Citation files from the searched databases were imported into Endnote reference management software and duplicates were removed. Using the eligibility criteria, two investigators independently screened titles and abstracts of the studies for relevance. The potential full texts articles were further assessed to be included in the review. Any disagreements between the authors were resolved with a discussion. Inclusion criteria were any observational or experimental studies that investigated both endometriosis and irritable bowel syndrome. Studies were included if irritable bowel syndrome was diagnosed by pre established criteria. Endometriosis had to be confirmed surgically, by clinical inspection, or reported as the International Classification of Diseases code for endometriosis. Meta-analyses, reviews, conference summaries, abstracts, case reports, opinions, letters, and animal studies were excluded. There was no search restriction for year of publication or the age group of patients. Articles were restricted to English and Spanish.

### Data extraction and quality assessment

Data were extracted into a standardized data-collection sheet using the following headings: first author name, date of publication, study site, study design, irritable bowel syndrome diagnosis criteria, endometriosis diagnostic criteria, sample size, event rate, and quality assessment score. Two investigators (MN and PR) assessed the quality of all included studies using the Newcastle-Ottawa Scale (NOS), and the overall scores were recorded (14). NOS scale is widely used for assessing quality of each included study in meta-analyses and is based on ranking studies on according to the selection criteria, group comparability and ascertainment of exposure.

### Data synthesis and analysis

Forest plots were generated with Review Manager version 5.4 (Nordic Cochrane Centre, Cochrane Collaboration, Denmark) and funnel plots were created with JASP statistical software. The primary outcome of the association of irritable bowel syndrome and endometriosis in this meta-analysis was performed using the random effects model to produce odds ratios (OR) with 95% confidence interval (CI). We conducted subgroup analyses based on diagnosis of endometriosis (surgical versus ICD-9-CM 617.x codes), method of diagnosis of irritable bowel syndrome, NOS scores (>6 vs. <6), and a combination of all criteria (endometriosis diagnosis; irritable bowel syndrome criteria; NOS score; and study design). In the subgroup analysis based on all criteria, studies were grouped as having met all criteria (surgical diagnosis of endometriosis, irritable bowel syndrome diagnosed with Rome criteria (15–17), NOS score > 6, and longitudinal studies), or not. This allowed for strong epidemiological evidence for the association between endometriosis and irritable bowel syndrome.

According to Rome III criteria, IBS patients can be classified into four subtypes and can be useful for treating specific symptoms of the patient. The subtypes include: IBS with diarrhea (IBS-D), IBS with constipation (IBS-C), IBS with mixed features (IBS-M) or IBS, unsubtyped. Whereas Rome IV criteria defined IBS as a functional bowel disorder in which recurrent abdominal pain is associated with defecation or a change in bowel habits.

A separate forest plot was generated for the prevalence of irritable bowel syndrome in patients with endometriosis, for studies that provided only prevalence data. The random effects model account for between-study heterogeneity by weighting studies similarly. Heterogeneity was assessed using the I^2^ statistic. Values of I^2^ > 50% were considered as indicative of large heterogeneity (18). We used the Begg’s and Egger’s funnel plot, which is a subjective visual method, to estimate risk of publication bias. A funnel plot that appears asymmetrical suggests publication bias. A *p*-value of <0.05 for all analyses was considered statistically significant. Although *p*-values are poor predictors of outcome, all quantitative studies included in our analyses mention *p*-values in accordance to the AM Stat recommendation.

## Results

### Search results and study inclusion

**FIGURE 1:**
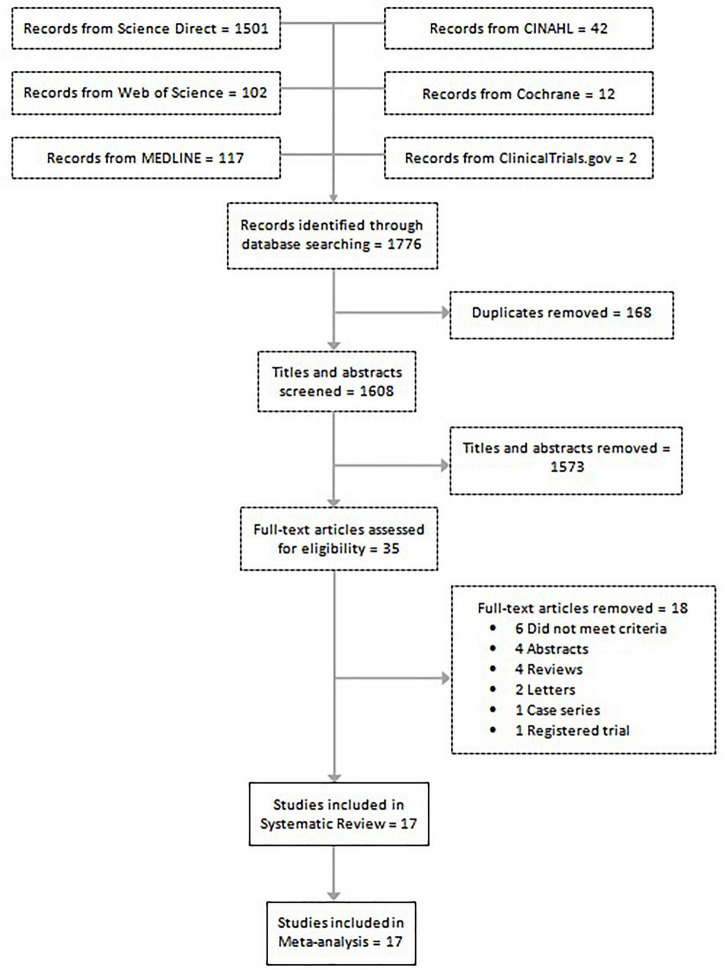
Flow diagram shows the included studies for the systematic review and meta-analysis on endometriosis and irritable bowel syndrome.

A total of 1,776 studies were identified in 6 separate databases. After removal of 168 duplicates, there were 1,608 eligible studies (titles/abstracts) which were independently screened by two reviewers. Of the 1,608 screened studies, 1,573 did not meet inclusion criteria and 35 full-text articles were reviewed. A total of 17 studies met criteria to be included in the systematic review. Eighteen studies were excluded for the following reasons: did not meet criteria; conference abstracts; reviews; letters; case series; and registered trials. The meta-analysis of the association of endometriosis and irritable bowel syndrome included 11 studies, and the meta-analysis on the prevalence of irritable bowel syndrome in endometriosis included 6 studies (see flow chart in Figure 1).

### Study characteristics

Overall 96,119 subjects were included in the main meta-analysis (11 studies) for endometriosis and irritable bowel syndrome association, with 18,887 endometriosis patients and 77,171 controls (patients without symptoms). The participants in the study by Ballard et al. (19) were already reported in the study by Seaman et al. (2), thus they were not added to the main meta-analysis twice. The meta-analysis on the prevalence of irritable bowel syndrome in endometriosis included 6,395 subjects. Almost all articles were published during the last decade, with two exceptions, which were published in 2005 and 2008. Of the 17 studies in this review, the majority were conducted in the United States, the United Kingdom, and Sweden. Table 1 describes the key characteristics of the included studies. Most studies used either Rome II (15), Rome III (16), Rome IV (17), or the visual analog scale for irritable bowel syndrome (VAS-IBS) (20) questionnaires to diagnose irritable bowel syndrome. Endometriosis diagnosis was confirmed either by laparoscopy or laparotomy. Each study had a quality assessment score between 5 and 10 on the Newcastle-Ottawa Scale (14), with most studies having a score of 7 or greater.

**TABLE 1:** Included study characteristics.

Study	Study site	Study design	IBS criteria	Endometriosis diagnosis	Sample size	OR/Prevalence	NOS score
**Association of endometriosis and IBS**							
Remorgida et al. (22)	Italy	Interventional	Rome II	Surgical	362	1.60	10
Seaman et al. (2)	United Kingdom	Case-control	History	Laparoscopy	26,779	3.50	7
Mamdouh et al. (23)	Egypt	Case-control	Not Stated	Laparoscopy	330	2.00	10
Issa et al. (24)	United Kingdom	Interventional	Rome III	Laparoscopy	80	2.03	7
Wu et al. (21)	Taiwan	Retrospective cohort	Not Stated	ICD-9-CM 617.x	36,456	1.95	7
Moore et al. (1)	New Zealand	Retrospective cohort	Rome III	Laparoscopy	231	3.02	6
Ek et al. (26)	Sweden	Case-cohort	VAS-IBS	Laparoscopy	289	1.63	6
Schomacker et al. (25)	Denmark	Cross-sectional	Rome III	Laparoscopy	356	4.35	7
Surrey et al. (3)	United States	Retrospective cohort	Not Stated	ICD-9-CM 617.x	30,705	2.90	5
Schink et al. (27)	Germany	Case-control	Not Stated	Laparoscopy	208	3.68	7
DiVasta et al. (10)	United States	Retrospective cohort	Rome IV	Laparoscopy	323	5.26	7
**Prevalence of IBS in endometriosis**							
Ballard et al. (19) Meurs-Sjozd et al. (30)	United Kingdom Netherlands	Case-control Prospective cohort	History Rome III	Laparoscopy Laparoscopy	5,540* 101	10.6%15%	76
Droz and Howard (28)	United States	Retrospective cohort	Rome II	Surgical	108	31%	8
Smorgick et al. (31)	United States	Retrospective cohort	Not stated	Surgical	164	17%	8
Ek et al. (29)	Sweden	Case-cohort	VAS-IBS	Laparoscopy	109	15%	6
Lee et al. (6)	Canada	Prospective cohort	Rome III	Laparoscopy	373	52%	8

IBS, inflammatory bowel syndrome; OR, odds ratio; NOS, Newcastle-Ottawa scale. *Reported cases in the study by Seaman et al. (2).

### Meta-analysis of studies

**FIGURE 2:**
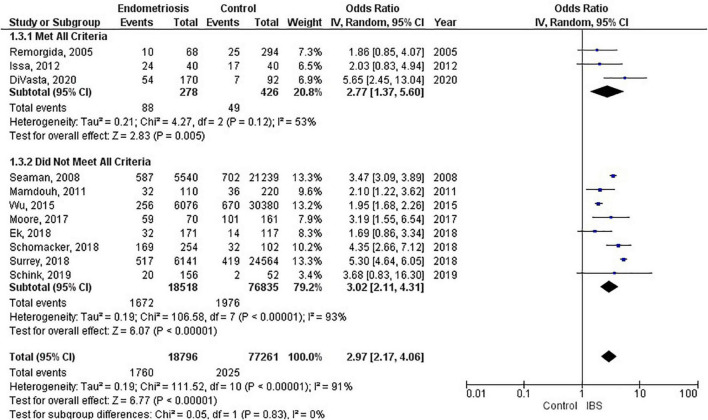
Forest plot by all criteria.

**FIGURE 3:**
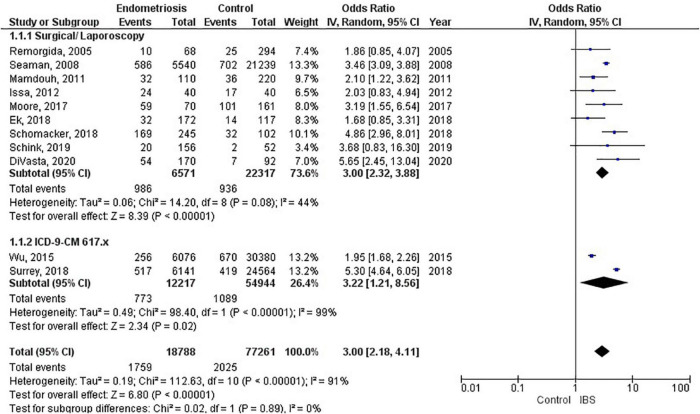
Forest plot by endometriosis diagnosis.

**FIGURE 4:**
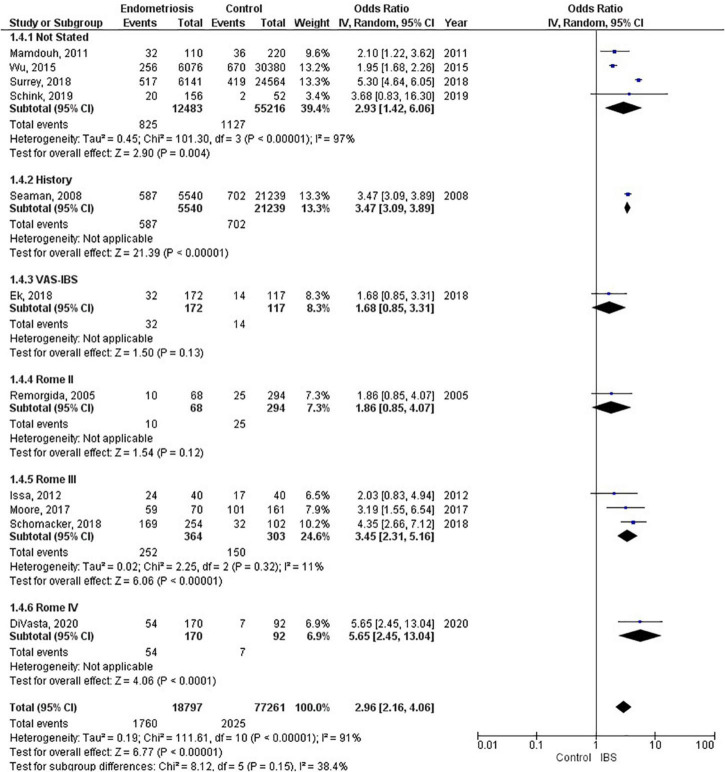
Forest plot by IBS criteria.

**FIGURE 5:**
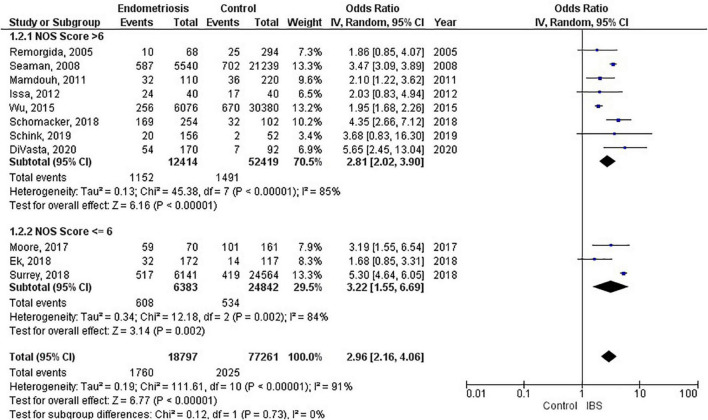
Forest plot by NOS score.

Of the 11 studies in the main meta-analysis (1–3, 10, 21–27), most were cohort and case control. Studies were conducted from 2005 to 2020, and sample sizes ranged from 80 to 36,456. In this meta-analysis the pooled odds ratio of endometriosis and irritable bowel syndrome was 2.97 (95% CI = 2.17 – 4.06), based on all selected criteria (see details in Figure 2). Odds ratio for the individual studies ranged from 1.69 (26) to 5.65 (10). There was a large heterogeneity in this study (I^2^ = 91%, [*P* < 0.00001]). In our subgroup analyses, the odds ratio for each subgroup was approximately 3, regarding endometriosis diagnosis (see Figure 3), criteria used for irritable bowel syndrome (see Figure 4), and NOS score (see Figure 5). Visual inspection of the funnel plot appears asymmetrical, suggesting the presence of publication bias (see Figure 7).

**FIGURE 6:**
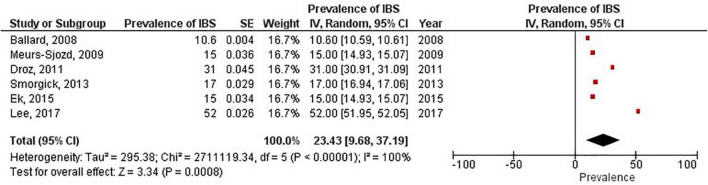
Forest plot by prevalence of IBS.

**FIGURE 7:**
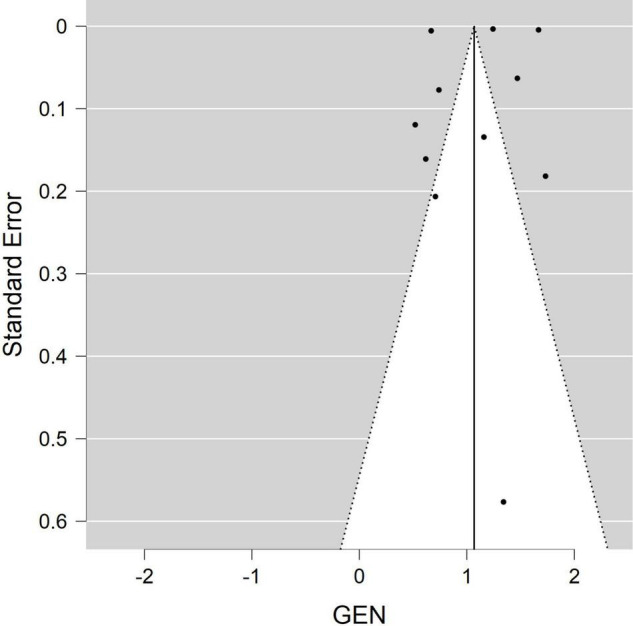
Funnel plot.

### Endometriosis diagnosis

There were 9 studies (28,888 patients) in the meta-analysis that confirmed endometriosis surgically, and 2 studies (67,161 patients) that used the ICD-9-CM 617.x codes to diagnosis endometriosis. The random effects model showed a significant association between endometriosis and irritable bowel syndrome with a pooled odds ratio of 3.0 (95% CI = 2.18, 4.11) (see Figure 3).

### Irritable bowel syndrome diagnostic criteria

Four studies (67,699 patients) did not state what criteria were used to diagnose irritable bowel syndrome, three studies (667 patients) used the Rome III criteria, and one study each used the following as their criteria: history (26,779 patients); VAS-IBS (289 patients); Rome II (362 patients), and Rome IV (323 patients). The random effects model shows a significant association between endometriosis and irritable bowel syndrome, with a combined odds ratio of 2.96 (95% CI = 2.16, 4.06) (see Figure 4).

### Pooled prevalence of irritable bowel syndrome

There were six studies (6, 19, 28–31) that estimated the prevalence of irritable bowel syndrome in women with endometriosis. These studies ranged in sample size from 101 to 5,540. The prevalence of irritable bowel syndrome in patients with endometriosis ranged from 10.6 to 52%, with a pooled estimate of 23.4% (95% CI = 9.7%, 37.2%) (see Figure 6).

## Discussion

This systematic review and meta-analysis was designed to estimate the association of endometriosis and irritable bowel syndrome. Our literature search included all available original studies investigating irritable bowel syndrome and endometriosis, thereby allowing us to include a large number of subjects (17 studies; *n* = 96,974).

The most significant finding of our study is that the pooled analysis showed endometriosis was associated with an almost three-fold increase in risk of irritable bowel syndrome, and that more than 1 in 5 women with endometriosis have irritable bowel syndrome. Of particular significance, all 11 studies in the main meta-analysis showed a positive association of irritable bowel syndrome and endometriosis. Moreover, almost all subjects in this analysis were followed longitudinally, either retrospectively or prospectively, thus allowing for inference on temporality vis-à-vis risk factor and disease. In addition, five studies (*n* = 68,129) included in this meta-analysis showed a positive association of endometriosis and irritable bowel syndrome, even after adjustments were made for potential confounding variables (3, 10, 21, 25, 26). Furthermore, there were significant findings in our subgroup analyses based on diagnostic method for endometriosis, diagnostic criteria for irritable bowel syndrome, and NOS scores. More importantly, after the studies in the main meta-analysis were categorized based on the following criteria: longitudinal study design; surgical confirmation of endometriosis; Rome diagnostic criteria for irritable bowel syndrome; and NOS scores > 6, the pooled odds ratio was 2.77 (95% CI = 1.37, 5.60). Thus, this provides strong epidemiological evidence for the increased association of endometriosis and irritable bowel syndrome.

Endometriosis is characterized as a chronic, estrogen-dependent inflammatory disorder with the presence of endometrial tissue outside the uterine cavity (3). Affected areas encompass the pelvic peritoneum, ovaries, rectovaginal septum, the abdominal cavity, and the gastrointestinal tract.

Histologically, endometriosis can be characterized into superficial endometriosis, ovarian endometrioma (OE) and deep infiltrating endometriosis (DIE). DIE can present with sever symptoms as the lesions penetrate deeper into the peritoneum and thus produce more pain as compared to the superficial. DIE also tends to involve the uterine ligaments, pouch of Douglas, rectum, or vagina. OE on the other hand, is the most common type of endometriosis and located in the pelvic areas or along the intestines. Multifocality of such a variably distributed lesion thus, predisposes to a variable clinical presentation the patients.

The relationship between endometriosis and irritable bowel syndrome has not yet been fully elucidated, and multiple theories have been proposed. One such theory is the immunological linkage through increased mast cell activation seen in both conditions (32). The major hallmarks postulated in this immunological linkage are the abnormal levels of inflammatory cytokines and immune cell activation in the peritoneal cavity (33). Retrograde menstruation has been a plausible explanation, which causes the dissemination of menstrual blood containing endometrial cells into the pelvic cavity, thus triggering symptoms of irritable bowel syndrome (34). Specifically, in endometriosis, the activated mast cells have been activated near nerve endings within the pelvic and abdominal regions, and in irritable bowel syndrome they have been shown to be activated near the bowel mucosa (35). Moreover, Remorgida et al. (22) have found that the severity of gastrointestinal symptoms was directly related to the extent of infiltration of endometriotic foci in the bowel, and reversal of symptoms occurred after removal of those lesions. However, they did not find any conclusive evidence regarding endometriosis and predisposition to a specific subtype of irritable bowel syndrome.

Another theory for the increased association between these two disorders is through a hormonal linkage. This hormonal connection involves the presence of gonadotropin releasing hormone-containing neurons (36) and receptors for luteinizing hormone within the pelvic organs (37) and the enteric nervous system (38). It is hypothesized that the pain experienced in some women with irritable bowel syndrome could be as a result of female sex hormones, as reports have shown a fluctuating exacerbation of symptoms of irritable bowel syndrome during menstruation (39). Likewise, it was observed that patients with endometriosis had worsening of gastrointestinal symptoms during the time of menstruation (30). It is posited that patients with endometriosis and irritable bowel syndrome both experience visceral hypersensitivity, which is likely to contribute to the severity of gastrointestinal symptoms (24). A large population-based study reported that the highest prevalence rate for endometriosis was for the 40–44-year age group (40), and Oka et al. reported that women between the ages of 30–39 years were more likely to have irritable bowel syndrome when compared to women less than 30 years old (8). Thus, the prevalence for both conditions peak at approximately the same age range, just around the beginning of the menopausal period. Moreover, postmenopausal women with irritable bowel syndrome experience symptoms more severely than premenopausal women with irritable bowel syndrome, most likely due to modulation in the brain-gut axis as a result of hormonal changes (41). Our study was not analyzed according the age of the patient.

Furthermore, a meta-analysis on the sex differences of irritable bowel syndrome reveals that women are more likely to experience abdominal pain when compared to men, and this may be because of sex hormonal differences (42).

Other important factors to consider when examining the relationship between endometriosis and irritable bowel syndrome are race/ethnicity and geographical region. In their study, Bougie et al. showed that Black women were less likely than White women to have endometriosis, and that Asian women were more likely than White women to have endometriosis (43). Similarly, Wigington et al. reported that Black women were less likely than White women to have irritable bowel syndrome (44). Thus, White women were more likely to have both endometriosis and irritable bowel syndrome when compared to Black women. Interestingly, of the 11 studies in our meta-analysis, only two studies stated the race of the participants (10, 27), and of these, the study by Schink investigated only Caucasian women (27).

As discussed previously, endometriosis is a chronic and multifactorial (genes, hormones, immune and environmental) and multi risk factor (family history, long menstrual cycle, low parity, and poor physical activity) associated disease (45, 46). An association between endometriosis and heavy metal sensitivity has been discussed in research that can potentially play a role in producing produce an IBS-like syndrome. Specifically, heavy metal nickel has been shown to interfere with estrogen and its receptors and thus plays a role in the pathogenesis of IBS. Researchers have even demonstrated a higher nickel level in endometriosis tissue (46, 47).

Recent global studies showed that the prevalence of irritable bowel syndrome varies from country to country, ranging from 0.2% in India to 29.2% in Croatia, using the Rome III criteria, and ranging from 0.4% in India and Ghana to 21.3% in United States, using the Rome IV criteria (8). Similarly, the global prevalence rates for endometriosis in the general population ranged from 0.7 to 8.6% (4). This highlights the importance of recognizing that irritable bowel syndrome and endometriosis can burden women of any race and from any country of origin, even though they can vary widely regarding presentation and response to treatment (43). Studies investigating endometriosis or irritable bowel syndrome individually were sparse for the geographical regions of South America, Central America, Africa, and Asia (8). However, the studies conducted in the United States reported the highest prevalence rate of endometriosis (48), and the highest prevalence rate of irritable bowel syndrome when using the Rome IV diagnostic criteria (11). Moreover, the studies conducted in the United States showed that women with endometriosis had the highest odds (5.65, 5.30) of having irritable bowel syndrome (see Figure 2). Thus, this points to further evidence that endometriosis is a significant contributory factor leading to irritable bowel syndrome. Needless to say, more investigation is needed regarding race/ethnicity and the association between endometriosis and irritable bowel syndrome.

Studies included in our meta-analysis used the Rome II, Rome III, and Rome IV criteria. The odds of irritable bowel syndrome in endometriosis increased with each subsequent updated version of the Rome criteria (odds ratio from 1.86 to 3.45 to 5.65), respectively. However, when interpreting these differences, one should also consider the significant heterogeneity that exists regarding study design and sample size. Moreover, recent studies have shown that the diagnostic outcomes for Rome II and Rome III criteria differ significantly (49), whereas there were comparable findings for Rome III and Rome IV criteria (50). Nevertheless, there is a markedly increased risk associated with endometriosis and irritable bowel syndrome, regardless of the criteria used to diagnose irritable bowel syndrome. The basis of the Rome criteria relies on its definition of irritable bowel syndrome in which recurrent abdominal pain is associated with defecation or a change in bowel habits (17). Thus, the Rome criteria classifies patients as different subtypes based on bowel habits: irritable bowel syndrome with predominant constipation (IBS-C), irritable bowel syndrome with predominant diarrhea (IBS-D), irritable bowel syndrome with mixed bowel habits (IBS-M) or irritable bowel syndrome, unclassified (IBS-U) (17). However, our data does not include information on these subtypes. Therefore, we cannot conclusively state whether endometriosis increases the risk of a specific subtype of irritable bowel syndrome over another, or if it increases the risk of all subtypes of irritable bowel syndrome.

Our meta-analysis included one study that used the visual analog scale for irritable bowel syndrome (VAS-IBS) to diagnose patients with irritable bowel syndrome. The VAS-IBS is a patient-centered questionnaire comprised of six categories: Abdominal Pain, Diarrhea, Constipation, Bloating and Flatulence, Abnormal bowel passage, and Vomiting and Nausea (20). The items in the VAS-IBS capture the main physical concerns women with irritable bowel syndrome might experience. All symptoms, except vomiting and nausea, support the diagnosis of irritable bowel syndrome. Even though the majority of studies in our meta-analysis used the Rome criteria to establish a diagnosis of irritable bowel syndrome, the VAS-IBS was shown to be an accurate and reliable questionnaire to diagnose irritable bowel syndrome (20).

Our literature search found two meta-analyses on endometriosis and irritable bowel syndrome (51, 52). Even though they had similar findings to ours regarding the increased association of endometriosis and irritable bowel syndrome, we believe that our review provides a more detailed analysis on various factors such as endometriosis diagnosis and irritable bowel syndrome diagnosis, and the pooled prevalence of irritable bowel syndrome in patients with endometriosis.

### Strengths and limitations

Strengths of this meta-analysis include incorporation of all available studies, with subsequent sub-analyses. Both observational and interventional studies were included. In addition, most studies included in the meta-analysis have a quality assessment rating greater than 6. Moreover, by independently reviewing articles and selecting those that fit our criteria, we concluded with a large-scale study from various geographic regions of the world that include North America, Europe, Asia, Africa, and Oceana. This allowed us to interpret the risk of irritable bowel syndrome in women with endometriosis from an extensive and multiethnic perspective. In addition, this is the first meta-analysis to include a pooled estimate of the prevalence of irritable bowel syndrome in women with endometriosis.

Our study has a number of potential limitations. While select studies employed either the Rome II, Rome III, Rome IV, or the visual analog scale for irritable bowel syndrome (VAS-IBS) as their criteria to gather symptomatic data on irritable bowel syndrome, the majority of studies in this meta-analysis did not state what criteria were used to diagnose irritable bowel syndrome. The anatomical location of endometriosis and the IBS subtypes was not described as relevant description was not available in the included studies. Nonetheless, after subgroup analysis by whether criteria was used or not, pooled estimates revealed similar results in these groups. These estimates were also reflected in the overall combined odds ratio for all studies. Thus, omitting the criteria used for establishing irritable bowel syndrome did not pose any significant error in this analysis. Another limitation of this study is that data from the two largest retrospective cohort studies identified patients with endometriosis using International Classification of Diseases, Ninth Revision, Clinical Modification (ICD-9-CM) 617.x codes, whereas all other studies stated that laparoscopy/laparotomy/clinical inspection was used as the mode of diagnosis. There is a significant variation in clinical diagnosis of endometriosis due to the costs and invasive diagnostic techniques including laparoscopic or surgical diagnosis. This has led to more reliance on radiological diagnosis for the same. Nevertheless, the surgical diagnostic methods are still considered the gold standard. Additionally, IBS diagnostic criteria are not based on standard guidelines or criteria. Most commonly used are the Manning and the Rome criteria which are possibly too general and vague for a specific diagnosis. Thus, an inevitable overlap occurs in the diagnosis of endometriosis and IBS (53).

Therefore, there was some inconsistency regarding identification of endometriosis. Nevertheless, our subgroup analysis regarding endometriosis diagnosis showed similar pooled estimates. However, despite these limitations, the diagnosis of irritable bowel syndrome remains a challenge with the fluctuation in symptoms and its symptoms mimicking other disorders like endometriosis (17).

### Recommendations

Our database search revealed that no studies were conducted in Central America or South America, and only a solitary study each arose out of Africa and Asia. Thus, we recommend that studies be conducted in these regions of the world to give globally representative estimates of the risk associated with these conditions. Furthermore, since the majority of participants were investigated in retrospective cohort studies, we recommend that researchers conduct large-scale prospective cohort studies to investigate the risk of irritable bowel syndrome (preferably using the Rome IV criteria) in women with endometriosis (with diagnosis confirmed surgically). Moreover, we suggest that studies be conducted to investigate whether endometriosis predisposes to any specific subtypes of irritable bowel syndrome.

## Conclusion

This review provides significant epidemiological evidence for the association between endometriosis and irritable bowel syndrome. Women with endometriosis are three times more likely to have irritable bowel syndrome compared to women without endometriosis. Doctors should be mindful that patients with endometriosis can also have irritable bowel syndrome.

## Data availability statement

The original contributions presented in this study are included in the article/supplementary material, further inquiries can be directed to the corresponding author.

## Author contributions

MN, MR, and SL contributed to the conception and design of the study. ME organized the database. PR and SN performed the statistical analysis. AV wrote the first draft of the manuscript. MN, MR, AV, and ME wrote the sections of the manuscript. All authors contributed to manuscript revision, read, and approved the submitted version.

## Footnotes

1
http://www.crd.york.ac.uk/PROSPERO/

## References

[1] Moore JS Gibson PR Perry RE Burgell RE. Endometriosis in patients with irritable bowel syndrome: specific symptomatic and demographic profile, and response to the low FODMAP diet. *Aust N Z J Obstet Gynaecol.* (2017) 57:201–5. 10.1111/ajo.12594 28303579

[2] Seaman HE Ballard KD Wright JT de Vries CS. Endometriosis and its coexistence with irritable bowel syndrome and pelvic inflammatory disease: findings from a national case-control study–Part 2. *BJOG.* (2008) 115:1392–6. 10.1111/j.1471-0528.2008.01879.x 18715239

[3] Surrey ES Soliman AM Johnson SJ Davis M Castelli-Haley J Snabes MC. Risk of developing comorbidities among women with endometriosis: a retrospective matched cohort study. *J Womens Health (Larchmt).* (2018) 27:1114–23. 10.1089/jwh.2017.6432 30070938

[4] Ghiasi M Kulkarni MT Missmer SA. Is endometriosis more common and more severe than it was 30 years ago? *J Minim Invasive Gynecol.* (2020) 27:452–61. 10.1016/j.jmig.2019.11.018 31816389

[5] Maddern J Grundy L Castro J Brierley SM. Pain in endometriosis. *Front Cell Neurosci.* (2020) 14:590823. 10.3389/fncel.2020.590823 33132854 PMC7573391

[6] Lee CE Yong PJ Williams C Allaire C. Factors associated with severity of irritable bowel syndrome symptoms in patients with endometriosis. *J Obstet Gynaecol Can.* (2018) 40:158–64. 10.1016/j.jogc.2017.06.025 28870721

[7] Saha L. Irritable bowel syndrome: pathogenesis, diagnosis, treatment, and evidence-based medicine. *World J Gastroenterol.* (2014) 20:6759–73. 10.3748/wjg.v20.i22.6759 24944467 PMC4051916

[8] Oka P Parr H Barberio B Black CJ Savarino EV Ford AC. Global prevalence of irritable bowel syndrome according to Rome III or IV criteria: a systematic review and meta-analysis. *Lancet Gastroenterol Hepatol.* (2020) 5:908–17. 10.1016/S2468-1253(20)30217-X 32702295

[9] Lovell RM Ford AC. Global prevalence of and risk factors for irritable bowel syndrome: a meta-analysis. *Clin Gastroenterol Hepatol.* (2012) 10:712.e–21.e. 10.1016/j.cgh.2012.02.029 22426087

[10] DiVasta AD Zimmerman LA Vitonis AF Fadayomi AB Missmer SA. Overlap between irritable bowel syndrome diagnosis and endometriosis in adolescents. *Clin Gastroenterol Hepatol.* (2021) 19:528–37.e1. 10.1016/j.cgh.2020.03.014 32184183

[11] Skoog SM Foxx-Orenstein AE Levy MJ Rajan E Session DR. Intestinal endometriosis: the great masquerader. *Curr Gastroenterol Rep.* (2004) 6:405–9. 10.1007/s11894-004-0058-6 15341718

[12] Vigano D Zara F Usai P. Irritable bowel syndrome and endometriosis: new insights for old diseases. *Dig Liver Dis.* (2018) 50:213–9. 10.1016/j.dld.2017.12.017 29396128

[13] Moher D Liberati A Tetzlaff J Altman DG Group P. Preferred reporting items for systematic reviews and meta-analyses: the PRISMA statement. *PLoS Med.* (2009) 6:e1000097. 10.1371/journal.pmed.1000097 19621072 PMC2707599

[14] Stang A. Critical evaluation of the Newcastle-Ottawa scale for the assessment of the quality of nonrandomized studies in meta-analyses. *Eur J Epidemiol.* (2010) 25:603–5. 10.1007/s10654-010-9491-z 20652370

[15] Thompson WG Longstreth GF Drossman DA Heaton KW Irvine EJ Muller-Lissner SA. Functional bowel disorders and functional abdominal pain. *Gut.* (1999) 45(Suppl. 2):II43–7. 10.1136/gut.45.2008.ii43 10457044 PMC1766683

[16] Longstreth GF Thompson WG Chey WD Houghton LA Mearin F Spiller RC. Functional bowel disorders. *Gastroenterology.* (2006) 130:1480–91. 10.1053/j.gastro.2005.11.061 16678561

[17] Lacy BE Patel NK. Rome criteria and a diagnostic approach to irritable bowel syndrome. *J Clin Med.* (2017) 6:99. 10.3390/jcm6110099 29072609 PMC5704116

[18] Grant J Hunter A. Measuring inconsistency in knowledgebases. *J Intell Inf Syst.* (2006) 27:159–84. 10.1007/s10844-006-2974-4

[19] Ballard KD Seaman HE de Vries CS Wright JT. Can symptomatology help in the diagnosis of endometriosis? Findings from a national case-control study–Part 1. *BJOG.* (2008) 115:1382–91. 10.1111/j.1471-0528.2008.01878.x 18715240

[20] Bengtsson M Ohlsson B Ulander K. Development and psychometric testing of the Visual Analogue Scale for Irritable Bowel Syndrome (VAS-IBS). *BMC Gastroenterol.* (2007) 7:16. 10.1186/1471-230X-7-16 17475020 PMC1868742

[21] Wu CY Chang WP Chang YH Li CP Chuang CM. The risk of irritable bowel syndrome in patients with endometriosis during a 5-year follow-up: a nationwide population-based cohort study. *Int J Colorectal Dis.* (2015) 30:907–12. 10.1007/s00384-015-2218-6 25916604

[22] Remorgida V Ragni N Ferrero S Anserini P Torelli P Fulcheri E. The involvement of the interstitial Cajal cells and the enteric nervous system in bowel endometriosis. *Hum Reprod.* (2005) 20:264–71. 10.1093/humrep/deh568 15576386

[23] Mamdouh HM Mortada MM Kharboush IF Abd-Elateef HA. Epidemiologic determinants of endometriosis among Egyptian women: a hospital-based case-control study. *J Egypt Public Health Assoc.* (2011) 86:21–6. 10.1097/01.EPX.0000395322.91912.56 21527837

[24] Issa B Onon TS Agrawal A Shekhar C Morris J Hamdy S Visceral hypersensitivity in endometriosis: a new target for treatment? *Gut.* (2012) 61:367–72. 10.1136/gutjnl-2011-300306 21868492

[25] Schomacker ML Hansen KE Ramlau-Hansen CH Forman A. Is endometriosis associated with irritable bowel syndrome? A cross-sectional study. *Eur J Obstet Gynecol Reprod Biol.* (2018) 231:65–9. 10.1016/j.ejogrb.2018.10.023 30326376

[26] Ek M Roth B Nilsson PM Ohlsson B. Characteristics of endometriosis: a case-cohort study showing elevated IgG titers against the TSH receptor (TRAb) and mental comorbidity. *Eur J Obstet Gynecol Reprod Biol.* (2018) 231:8–14. 10.1016/j.ejogrb.2018.09.034 30317144

[27] Schink M Konturek PC Herbert SL Renner SP Burghaus S Blum S Different nutrient intake and prevalence of gastrointestinal comorbidities in women with endometriosis. *J Physiol Pharmacol.* (2019) 70:255–68. 10.26402/jpp.2019.2.09 31443088

[28] Droz J Howard FM. Use of the short-form McGill pain questionnaire as a diagnostic tool in women with chronic pelvic pain. *J Minim Invasive Gynecol.* (2011) 18:211–7. 10.1016/j.jmig.2010.12.009 21354067

[29] Ek M Roth B Ekstrom P Valentin L Bengtsson M Ohlsson B. Gastrointestinal symptoms among endometriosis patients–a case-cohort study. *BMC Womens Health.* (2015) 15:59. 10.1186/s12905-015-0213-2 26272803 PMC4535676

[30] Meurs-Szojda MM Mijatovic V Felt-Bersma RJ Hompes PG. Irritable bowel syndrome and chronic constipation in patients with endometriosis. *Colorectal Dis.* (2011) 13:67–71. 10.1111/j.1463-1318.2009.02055.x 19832874

[31] Smorgick N Marsh CA As-Sanie S Smith YR Quint EH. Prevalence of pain syndromes, mood conditions, and asthma in adolescents and young women with endometriosis. *J Pediatr Adolesc Gynecol.* (2013) 26:171–5. 10.1016/j.jpag.2012.12.006 23507008

[32] Barbara G Stanghellini V De Giorgio R Cremon C Cottrell GS Santini D Activated mast cells in proximity to colonic nerves correlate with abdominal pain in irritable bowel syndrome. *Gastroenterology.* (2004) 126:693–702. 10.1053/j.gastro.2003.11.055 14988823

[33] Laschke MW Menger MD. The gut microbiota: a puppet master in the pathogenesis of endometriosis? *Am J Obstet Gynecol.* (2016) 215:68.e1–4. 10.1016/j.ajog.2016.02.036 26901277

[34] Burney RO Giudice LC. Pathogenesis and pathophysiology of endometriosis. *Fertil Steril.* (2012) 98:511–9. 10.1016/j.fertnstert.2012.06.029 22819144 PMC3836682

[35] Anaf V Chapron C El Nakadi I De Moor V Simonart T Noel JC. Pain, mast cells, and nerves in peritoneal, ovarian, and deep infiltrating endometriosis. *Fertil Steril.* (2006) 86:1336–43. 10.1016/j.fertnstert.2006.03.057 17007852

[36] Hammar O Ohlsson B Veress B Alm R Fredrikson GN Montgomery A. Depletion of enteric gonadotropin-releasing hormone is found in a few patients suffering from severe gastrointestinal dysmotility. *Scand J Gastroenterol.* (2012) 47:1165–73. 10.3109/00365521.2012.706826 22835010

[37] Yung Y Aviel-Ronen S Maman E Rubinstein N Avivi C Orvieto R Localization of luteinizing hormone receptor protein in the human ovary. *Mol Hum Reprod.* (2014) 20:844–9. 10.1093/molehr/gau041 24874553

[38] Sand E Bergvall M Ekblad E D’Amato M Ohlsson B. Expression and distribution of GnRH, LH, and FSH and their receptors in gastrointestinal tract of man and rat. *Regul Pept.* (2013) 187:24–8. 10.1016/j.regpep.2013.09.002 24103690

[39] Meleine M Matricon J. Gender-related differences in irritable bowel syndrome: potential mechanisms of sex hormones. *World J Gastroenterol.* (2014) 20:6725–43. 10.3748/wjg.v20.i22.6725 24944465 PMC4051914

[40] Eisenberg VH Weil C Chodick G Shalev V. Epidemiology of endometriosis: a large population-based database study from a healthcare provider with 2 million members. *BJOG.* (2018) 125:55–62. 10.1111/1471-0528.14711 28444957

[41] Lenhart A Naliboff B Shih W Gupta A Tillisch K Liu C Postmenopausal women with irritable bowel syndrome (IBS) have more severe symptoms than premenopausal women with IBS. *Neurogastroenterol Motil.* (2020) 32:e13913. 10.1111/nmo.13913 32469130 PMC7529855

[42] Adeyemo MA Spiegel BM Chang L. Meta-analysis: do irritable bowel syndrome symptoms vary between men and women? *Aliment Pharmacol Ther.* (2010) 32:738–55. 10.1111/j.1365-2036.2010.04409.x 20662786 PMC2932820

[43] Bougie O Yap MI Sikora L Flaxman T Singh S. Influence of race/ethnicity on prevalence and presentation of endometriosis: a systematic review and meta-analysis. *BJOG.* (2019) 126:1104–15. 10.1111/1471-0528.15692 30908874

[44] Wigington WC Johnson WD Minocha A. Epidemiology of irritable bowel syndrome among African Americans as compared with whites: a population-based study. *Clin Gastroenterol Hepatol.* (2005) 3:647–53. 10.1016/s1542-3565(05)00367-8 16206496

[45] Parazzini F Esposito G Tozzi L Noli S Bianchi S. Epidemiology of endometriosis and its comorbidities. *Eur J Obstetr Gynecol Reproduct Biol.* (2017) 209:3–7. 10.1016/j.ejogrb.2016.04.021 27216973

[46] Borghini R Porpora MG Casale R Marino M Palmieri E Greco N Irritable Bowel syndrome-like disorders in endometriosis: prevalence of nickel sensitivity and effects of a low-nickel diet. An open-label pilot study. *Nutrients.* (2020) 12:341. 10.3390/nu12020341 32012984 PMC7071203

[47] Silva N Senanayake H Waduge V. Elevated levels of whole blood nickel in a group of Sri Lankan women with endometriosis: a case control study. *BMC Res Notes.* (2013) 6:13. 10.1186/1756-0500-6-13 23317102 PMC3548700

[48] Fuldeore MJ Soliman AM. Prevalence and symptomatic burden of diagnosed endometriosis in the United States: national estimates from a cross-sectional survey of 59,411 women. *Gynecol Obstet Invest.* (2017) 82:453–61. 10.1159/000452660 27820938

[49] Sperber AD Shvartzman P Friger M Fich A. A comparative reappraisal of the Rome II and Rome III diagnostic criteria: are we getting closer to the ‘true’ prevalence of irritable bowel syndrome? *Eur J Gastroenterol Hepatol.* (2007) 19:441–7. 10.1097/MEG.0b013e32801140e2 17489053

[50] Black CJ Craig O Gracie DJ Ford AC. Comparison of the Rome IV criteria with the Rome III criteria for the diagnosis of irritable bowel syndrome in secondary care. *Gut.* (2021) 70:1110–6. 10.1136/gutjnl-2020-322519 32973070

[51] Chiaffarino F Cipriani S Ricci E Mauri PA Esposito G Barretta M Endometriosis and irritable bowel syndrome: a systematic review and meta-analysis. *Arch Gynecol Obstet.* (2021) 303:17–25. 10.1007/s00404-020-05797-8 32949284

[52] Saidi K Sharma S Ohlsson B. A systematic review and meta-analysis of the associations between endometriosis and irritable bowel syndrome. *Eur J Obstet Gynecol Reprod Biol.* (2020) 246:99–105. 10.1016/j.ejogrb.2020.01.031 32004880

[53] Kwok H Jiang H Li T Yang H Fei H Cheng L Lesion distribution characteristics of deep infiltrating endometriosis with ovarian endometrioma: an observational clinical study. *BMC Womens Health.* (2020) 20:111. 10.1186/s12905-020-00974-y PMC7240912 32434535

